# Bioremediation of Crude Glycerol by a Sustainable Organic–Microbe Hybrid System

**DOI:** 10.3389/fmicb.2021.654033

**Published:** 2021-04-08

**Authors:** Ho Shing Chan, Kemeng Xiao, Tsz Ho Tsang, Cuiping Zeng, Bo Wang, Xingxing Peng, Po Keung Wong

**Affiliations:** ^1^School of Life Sciences, The Chinese University of Hong Kong, Hong Kong SAR, China; ^2^CAS Key Laboratory of Quantitative Engineering Biology, Shenzhen Institute of Synthetic Biology, Shenzhen Institutes of Advanced Technology, Chinese Academy of Sciences, Shenzhen, China; ^3^School of Environmental Science and Engineering, Sun Yat-sen University, Guangzhou, China; ^4^Institute of Environmental Health and Pollution Control, Guangdong University of Technology, Guangzhou, China

**Keywords:** hybrid system, crude glycerol, hydrogen production, *Klebsiella pneumoniae*, hydrothermal carbonation carbon

## Abstract

*Klebsiella pneumoniae* with crude glycerol-utilizing and hydrogen (H_2_)-producing abilities was successfully isolated from return activated sludge from Shatin Sewage Treatment Works. The H_2_ production strategy used in this study was optimized with crude glycerol concentrations, and 1,020 μmol of H_2_ was generated in 3 h. An organic–microbe hybrid system was constructed with metal-free hydrothermal carbonation carbon (HTCC) microspheres to enhance the H_2_ production under visible light (VL) irradiation. Under optimized VL intensity and HTCC concentration, an elevation of 35.3% in H_2_ production can be obtained. Electron scavenger study revealed that the photogenerated electrons (e^–^) from HTCC contributed to the additional H_2_ production. The variation in intercellular intermediates, enzymatic activity, and reducing equivalents also suggested that the photogenerated e^–^ interacted with *K. pneumoniae* cells to direct the metabolic flux toward H_2_ production. This study demonstrated the feasibility of using an organic–microbe hybrid system as a waste-to-energy technology.

## Introduction

Crude glycerol (CG) is the major by-product from the biodiesel industry through esterification and transesterification of triglycerides in vegetable oils and animal fats, and the amount produced is approximately 10% of biodiesel production (Garlapati et al., 2016). However, the CG produced is not pure, which contains organic salt, ash, soap, residual free fatty acid, and catalyst (Hunsom and Autthanit, 2013; Ardi et al., 2015; Luo et al., 2016; Rahman et al., 2017; Ma et al., 2019), and also, the glycerol content varies among different manufacturing companies. Conventional purification and refining processes of CG are expensive and energy-intensive, which deter the regeneration of pure glycerol; thus, the substantial production of CG is an environmental and financial liability of the biodiesel industry (Thompson and He, 2006; Ardi et al., 2015; Luo et al., 2016). Hence, the direct use of CG for the value-added process was investigated by many researchers. Many previous studies have demonstrated the bioconversion of CG by microorganism to convert CG into different metabolites (Garlapati et al., 2016), for example, hydrogen (H_2_) (Liu and Feng, 2007; Chookaew et al., 2012; Pott et al., 2014), ethanol (Yazdani and Gonzalez, 2008; Oh et al., 2011), and 1,3-propanediol (1,3-PDO) (Oh et al., 2008; Casali et al., 2012; Wilkens et al., 2012). Among these different metabolites, H_2_ is a potential renewable and sustainable energy source with nearly zero emissions of carbon and high energy capacity (Midilli and Dincer, 2008; Jain, 2009; Nicoletti et al., 2015; Chu et al., 2017).

Recently, different kinds of organic–biological hybrid systems have been developed for energy production. Brown et al. (2012) developed a system of CdS nanorods capped with hydrogenase purified from *Clostridium acetobutylicum* to produce H_2_ under light irradiation. However, even with high product selectivity, the purification complexity and inherent instability of the enzymes restricted the application of the enzyme-based systems. After that, cell-based biohybrid systems were developed. For example, Sakimoto et al. (2016) and Wang et al. (2017) successfully precipitated CdS nanoparticles on the surface of the *Moorella thermoacetica* and *Escherichia coli* to reduce CO_2_ and produce H_2_ under irradiation, respectively. More recently, Ramprakash and Incharoensakdi (2020) constructed another organic–biological system with TiO_2_ and *E. coli* cells. These cell-based hybrids not only produce a wide variety of products with the metabolic pathway of cells but also possess cellular self-replication and self-repairment mechanisms, therefore with potentially high scalability. Despite these strengths, challenges such as the leakage of the metal ions (Cd^2+^ and Ti^2+^) and complex fabrication method (biological precipitation of CdS) for the hybrid system impede their prospects for commercialization. In addition, the precursor of the *E. coli*–CdS hybrid system was glucose, which was relatively expensive and competed with the food industry. Therefore, it is essential to develop a biocompatible and cost-effective hybrid system for energy conversion with a more straightforward method, such as the direct mixing of photocatalyst with whole bacterial cells.

It has been reported that the non-metal graphitic carbon nitride (g-C_3_N_4_) has been combined with *Ralstonia eutropha* for bioplastic production under light irradiation (Xu et al., 2019). This proof-of-concept study illustrated that the organic photocatalysts could also enhance the productivity of the cell-based hybrid system with good biological compatibility. Similar to g-C_3_N_4_, the hydrothermal carbonation carbon (HTCC) is proven to be an effective photocatalyst with a narrow bandgap for light absorption and easily obtained *via* a simple hydrothermal treatment of carbohydrates such as glucose, starch, or even grass and wastes with relatively low cost and good biocompatibility (Hu et al., 2017; Wang et al., 2018). Therefore, the feasibility of HTCC for biohybrid study is worthy of study to mitigate the issues of secondary pollution caused by the introduction of metal-based photocatalysts. In addition, the *Klebsiella pneumoniae* was isolated from the activated sludge to utilize the CG for energy transformation. Compared with the previous bacterial species (*M. thermoacetica* and *E. coli*) applied in the hybrid system, the isolated *K. pneumoniae* is easy to cultivate with excellent environmental application without the need of complex genetic operation. Also, the use of the CG as precursors is much more environmentally friendly and cost-effective compared with that of glucose-based energy conversion reactions in the previously reported hybrid systems.

Therefore, in this work, for the first time, an organic–microbe hybrid system was developed with the combination of *K. pneumoniae* and HTCC to effectively convert CG into H_2_. The isolation and purification of a bacterial strain capable of utilizing CG and producing H_2_ were carried out. Also, the concentration of CG, visible light intensity, and HTCC concentration were optimized to achieve the best H_2_ efficiency. In the meanwhile, the mechanism for additional H_2_ generation was also studied.

## Materials and Methods

### Materials Preparation and Characterization

Crude glycerol was supplied by Champway Technology Limited and return activated sludge (RAS) collected from Shatin Sewage Treatment Works (SSTW), both of which were stored at 4°C. The CG was sterilized before use, and its composition is summarized in Supplementary Table 1.

The synthesis of HTCC was adopted from Hu et al. (2017). Briefly, 10 g of glucose was dissolved in 80 mL of ultrapure water in a 100 mL Teflon-lined stainless-steel autoclave. The hydrothermal reaction of the homogenous solution was performed at 180°C for 10 h. After cooling down to room temperature, the product was washed several times with ultrapure water and ethanol to remove the residual glucose and other impurities. It was dried at 60°C overnight before use.

The characterization of the as-prepared HTCC was then preformed. X-ray diffraction (XRD) pattern was recorded by a Rigaku SmartLab 9-kW X-ray diffractometer (Rigaku Corporation, Tokyo, Japan). The general morphology of the synthesized HTCC was studied using a Quanta 400 FEG MK2 scanning electron microscopr (SEM) (FEI Company, OR, United States). X-ray photoelectron spectroscopy (XPS) spectra of HTCC were recorded by a Thermo Nexsa X-Ray Photoelectron Spectrometer System (Thermo Fisher Scientific, Waltham, MA, United States). A PerkinElmer Lambda 950 UV/VIS/NIR Spectrometer (PerkinElmer Life and Analytical Sciences, Shelton, CT, United States) equipped with a 150-mm Integrating Sphere was used to record the Ultraviolet-Visible-Near infrared diffuse reflectance spectrum (UV-VIS-NIR DRS). CHI 760E Electrochemical Workstation (Shanghai Chen Hua Instrument Company, China) was used to measure photocurrent generation by HTCC under 2,000 W m^–2^ VL irradiation.

### Isolation and Identification of Crude Glycerol-Utilizing and Hydrogen-Producing Bacteria

Return activated sludge (0.1 mL) was spread on agar plates of 3-(N-morpholino)propanesulfonic acid (MOPS) medium (Supplementary Table 2; Neidhardt et al., 1974; Wang et al., 2017) supplemented with 20, 50, and 80 g L^–1^ CG. The plates were incubated at 30°C for 24 h to obtain isolated bacterial colonies. The pure colonies of isolated bacterial strains were separately inoculated in 50 mL of MOPS medium supplemented with 20 g L^–1^ CG aerobically at 30°C for 24 h with shaking at 160 rpm. After cultivation, the bacterial cells were centrifuged at 10,000 × *g* for 5 min and resuspended in 50 mL of BC medium (Supplementary Table 3) supplemented with 20 g L^–1^ CG, then cultured anaerobically at 30°C for 24 h in an anaerobic jar with an AnaeroGen sachet for hydrogenase induction. After that, the bacterial cells were harvested by centrifugation and resuspended in 50 mL of fresh simplified BC (SBC) medium (Supplementary Table 3) supplemented with 20 g L^–1^ CG, then transferred into a reactor and purged with nitrogen gas for 10 min to create an anaerobic environment for H_2_ production. A circulating water bath (SD07R-20, PolyScience, United States) was used to control the reaction temperature at 30°C, and magnetic stirring was applied to prevent the settling of bacteria. At a fixed time interval during the process of H_2_ production, a fixed volume of a 1-mL gaseous sample was collected. H_2_ was determined by a gas chromatograph (GC-7806, Shiweipx, Beijing, China) equipped with a 5-Å molecular sieve column and thermal conductivity detector using argon (Ar, purity ≥99.998%) as a carrier gas.

Fatty acid profiling was preformed using Sherlock Microbial Identification System (MIDI Inc., 2012, Newark, DE, United States). Briefly, the bacterium was first incubated on a trypticase soy broth agar plate at 30°C for 24 h. The bacterial cells were harvested and followed by saponification to liberate the fatty acids from the cellular lipids, then methylated to form fatty acid methyl esters. The fatty acid methyl esters were extracted from the aqueous phase to the organic phase and base-washed before analysis with gas chromatography. The 16S ribosomal RNA sequencing was performed by BGI Co., Ltd. Briefly, the bacterial genomic DNA was first extracted and followed by PCR amplification using universal primers, forward primer 27F (5′-AGAGTTTGATCMTGGCTCAG-3′) and reverse primer 1492R (5′-GGTTACCTTGTTACGACTT-3′). The amplified PCR products were analyzed by ABI 3730 sequencer, and the result was compared with BLASTn sequence database of the National Center for Biotechnology Information.

### Organic–Microbe Hybrid System for Hydrogen Production

An organic–microbe hybrid system for H_2_ production was constructed by direct mixing of bacterial suspension and HTCC under VL irradiation from Xenon lamp (PLS-SXE300D, Beijing PerfectLight) with a 420-nm cutoff filter. The VL intensity and HTCC concentration for H_2_ production by the hybrid system were optimized by varying light intensity from 0 to 3,000 W m^–2^ and HTCC concentration from 0 to 2 g L^–1^.

Stability of the hybrid system was studied by monitoring the cell viability, physiological activity and membrane integrity of *K. pneumoniae* cells. For cell viability, 1 mL aliquot of reaction mixture was serially diluted and 0.1 mL aliquots of each dilution were spread on nutrient agar plates. The plates were incubated at 30 °C for 24 h and the viable cell density was quantified by counting the number of CFU in the plates. For physiological activity, 2,3,5-triphenyltetraazolium chloride (TTC) dehydrogenase activity of *K. pneumoniae* cells was monitored (Klapwijk et al., 1974; Wang et al., 2017). One mL aliquot of reaction mixture was centrifuged at 10,000 × g for 3 min and resuspended with 1 mL SBC medium supplemented with 1% (w/v) TTC solution. The suspension was incubated at 37 °C for 2 h in dark. The red-colored product was extracted with 1 mL acetone under 30 min sonication and the absorbance was monitored at 484 nm using a UV-Vis spectrometer (BlueStar A, LabTech Group). For membrane integrity, intracellular potassium (K^+^) leakage was determined by measuring the K^+^ concentration of reaction mixture with inductively coupled plasma – optical emission spectrometer (ICP-OES, Optima 4300 DV ICP-OES system, PerkinElmer Inc., Wellesley, United States).

Biologically safe dosages of 0.05 and 0.2 mM chromium(VI) (Cr (VI)) were added as electron scavengers to parallelly prepare 50-mL bacterial cultures to quench the photogenerated e^–^. The photocatalytic water splitting was monitored by irradiating the reaction mixture of 1 g L^–1^ HTCC and SBC medium supplemented with 20 g L^–1^ CG and 10% (v/v) methanol. To determine the interaction between the photogenerated e^–^ and biological H_2_ production (BHP) pathway of the bacterium, the change in utilization efficiency of CG, the production of pyruvate, formate, lactate, and 1,3-PDO, and the hydrogenase activity, as well as reduced nicotinamide adenine dinucleotide (NADH)/nicotinamide adenine dinucleotide (NAD) ratio, were monitored in the hybrid system during the H_2_ production process. At fixed time intervals, 1 mL aliquots of the reaction mixture were collected for analysis.

Assay kits for pyruvate (MAK071, Sigma-Aldrich), formate (MAK059, Sigma-Aldrich), and lactate (MAK065, Sigma-Aldrich), as well as NADH/NAD (MAK037, Sigma-Aldrich), were used to quantify the amount of these intracellular compounds at fixed time intervals according to the procedures in the technical bulletins of the assay kits. The absorbance was measured using a 96-well plate reader (Spark 10-M multimode microplate reader, Tecan, Switzerland), and the concentrations of the individual substances were calculated using the corresponding standard curve.

The formate hydrogen lyase (FHL) activity of *K. pneumoniae* in the hybrid system was monitored by the method of formate-dependent reduction of benzyl viologen (BV) (Bagramyan et al., 2002) with modifications. Of the reaction mixture, 0.5 mL aliquots were collected at fixed time intervals and centrifuged at 10,000 × *g* for 10 min. The cell pellet was resuspended with a mixture of 1.84 mL of Tris-phosphate buffer (pH 7.5, supplemented with 5 × 10^–3^-M MgSO_4_), 80 uL of 1 M sodium formate, and 80 uL of 100 mM BV. The reaction mixture was quickly transferred into an anaerobic cuvette and incubated in 37°C water bath for 1 h. The absorbance of the reduced BV was monitored with UV–VIS spectrophotometer at 603 nm. The activity of FHL of the hybrid system was expressed in the percentage of the activity of the control group at the beginning of the experiment.

Residual CG and 1,3-PDO in the medium were monitored by high-performance liquid chromatography (Hewlett Packard 1100 Series HPLC System) coupled with a refractive index detector (Waters 410 Differential Refractometer) and an Aminex HPX- 87H column (300 mm × 7.8 mm, Bio-Rad, United States). The column temperature was set at 65°C, and the mobile phase was 5 mM H_2_SO_4_ with a flow rate of 0.8 mL/min.

## Results and Discussion

### Identification of Isolated Bacterial Strain and the Crude Glycerol Optimization for Hydrogen Production

**FIGURE 1:**
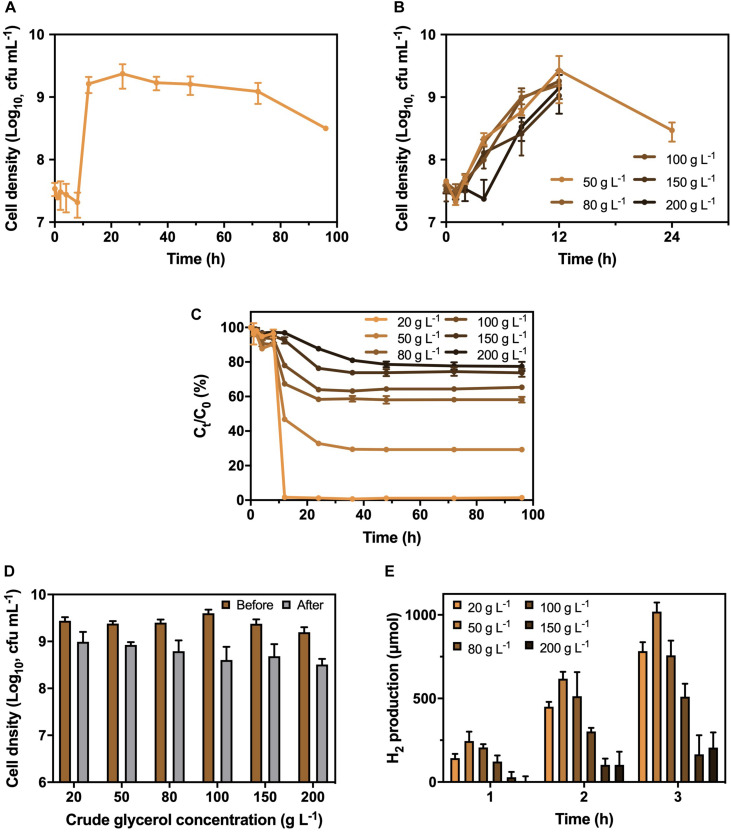
Growth pattern of *K. pneumoniae* **(A)** under 20 g L^−1^ CG and **(B)** 50 to 200 g L^−1^ CG, **(C)** corresponding residual CG in culture medium as well as **(D)** cell density and **(E)** H_2_ production in 3 h by *K. pneumoniae* after hydrogenase induction under different CG concentrations.

A bacterial strain with the abilities of CG utilization and H_2_ production was successfully isolated from RAS. The fatty acid profiling and 16S ribosomal RNA sequencing indicated that the isolated strain was identified as *K. pneumoniae* (Supplementary Table 4), with a rod shape under a microscope (Supplementary Figure 1). Besides, the isolated *K. pneumoniae* could produce approximately 780 μmol of H_2_ in 3 h (Supplementary Figure 2) with the supplementary CG. To optimize the CG concentration, the growth curve of *K. pneumoniae* under different CG concentrations ranged from 20 to 200 g L^−1^ was investigated (Figures 1A,B). The results showed that the growth of *K. pneumoniae* reached stationary phase after 12 h and remained stable at 9 Log_10_ cfu mL^−1^ for more than 60 h at 20 g L^−1^ CG. However, cell densities dropped below 6 Log_10_ cfu mL^−1^ after 24 h with the concentration of CG increasing from 50 to 200 g L^−1^, which was caused by the toxicity of impurities and osmotic pressure induced by the high concentration of CG. Meanwhile, *K. pneumoniae* could completely remove 20 g L^−1^ CG in culture medium within 12 h (Figure 1C). The synchronized trend of the bacterial exponential phase and decrease in CG concentration suggested that the *K. pneumoniae* can utilize CG as carbon source for growth. Therefore, 20 g L^−1^ of CG concentration was chosen to cultivate the *K. pneumoniae*.

Before the H_2_ production, the *K. pneumoniae* was anaerobically cultured in BC medium supplemented with different CG concentrations to induce the hydrogenase. Therefore, the optimal CG concentration for hydrogenase induction was also performed (Figure 1D). As displayed in Figure 1D, the bacterial density was similar after hydrogenase induction under CG concentration of 20 to 50 g L^−1^, while it reduced under increasing CG concentration from 80 to 200 g L^−1^ with toxic impurities and high osmotic pressure. Besides, the H_2_ production efficiency of *K. pneumoniae* after hydrogenase induction at different CG concentrations was examined in SBC medium supplemented with 20 g L^−1^ CG (Figure 1E). Bacterial culture with hydrogenase induction at 50 g L^−1^ CG showed the highest H2 production efficiency of 1,020 μmol in 3 h. Relatively low H_2_ production was observed for *K. pneumoniae* cells undergone hydrogenase induction at 150 to 200 g L^−1^ CG containing large number of impurities. Hence, 50 g L^−1^ CG was selected as the optimal concentration for hydrogenase induction.

**FIGURE 2:**
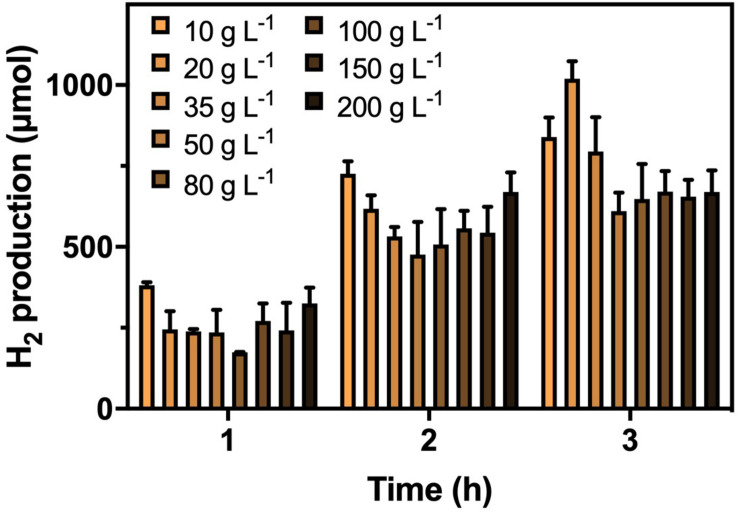
H_2_ production by *K. pneumoniae* under different CG concentrations after optimized cultivation and hydrogenase induction in 3 h.

After confirming the optimal CG concentrations for cultivation and hydrogenase induction, the H_2_ production efficiency of *K. pneumoniae* at CG concentrations of 20–200 g L^–1^ was performed (Figure 2). Highest H_2_ production of 1,020 μmol in 3 h was obtained under 20 g L^–1^ CG, and thus, it was considered the optimal concentration for H_2_ production. Interestingly, the H_2_ production was restricted to a certain level of approximately 650 μmol at high CG concentrations regardless of the actual amount of CG. This was caused by the flexibility of glycerol metabolism of *K. pneumoniae* leading to a higher H_2_ production efficiency at 20 g L^–1^ CG, whereas restricted H_2_ production was observed at a higher concentration of CG (Streekstra et al., 1987; Zeng et al., 1993, 1996). Two sublevels of CG concentration, 10 and 35 g L^–1^, were also applied to confirm the optimal concentration of 20 g L^–1^. For 10–20 g L^–1^ CG, H_2_ production increased with CG concentration, whereas the amount of produced H_2_ decreased from 20 g L^–1^ to a higher CG concentration. The major reason is that the CG concentration below 20 g L^–1^ was fallen into the glycerol-limiting condition, and so the production of H_2_ is proportional to the amount of CG (Zeng et al., 1993). Therefore, 20 g L^–1^ of CG concentration was optimized for the H_2_ production in the SBC medium.

### Characterization of Hydrothermal Carbonation Carbon

**FIGURE 3:**
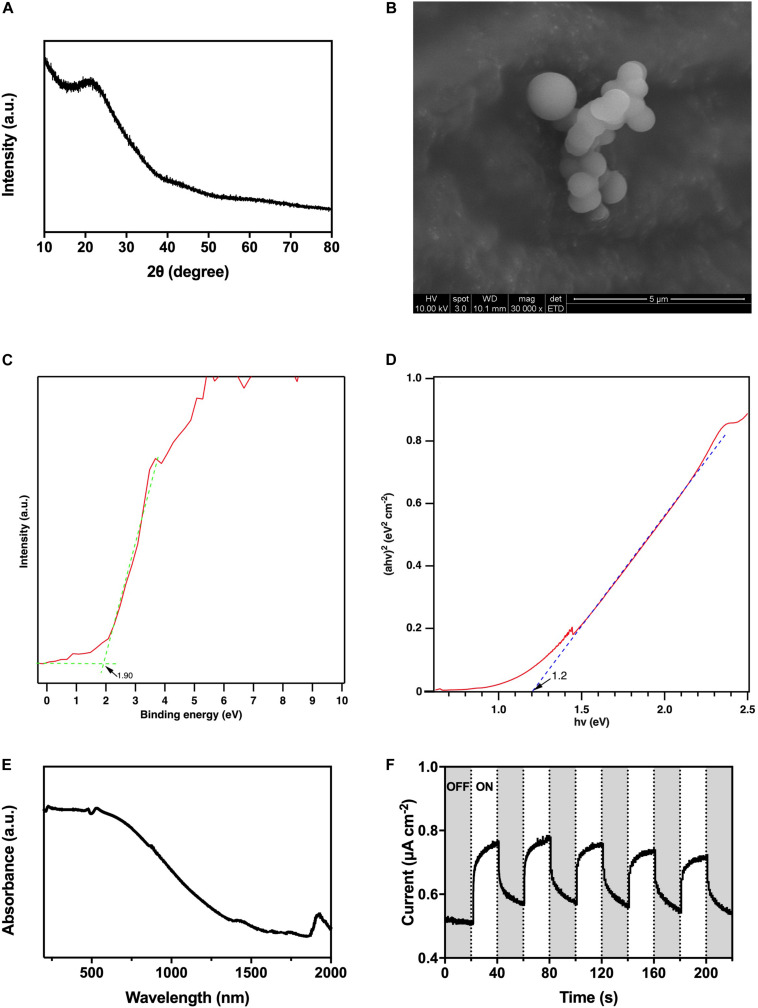
Characterization of HTCC, **(A)** X-ray diffraction pattern, **(B)** scanning electron microscope images, **(C)** valence band X-ray photoelectron spectroscopy spectrum, **(D)** converted Kubelka–Munk plot, **(E)** ultraviolet–visible–near infrared diffuse reflectance spectrum, and **(F)** photocurrent responses under 2,000 W m^− 2^ VL irradiation.

According to the XRD pattern (Figure 3A), there was no observable strong distinctive peak, so the as-prepared HTCC particles were amorphous. From the SEM image (Figure 3B), the HTCC particles were microspheres with 1-μm diameter.

The UV-VIS-NIR DRS revealed that the HTCC powders exhibited a strong absorption of UV/VIS irradiation, and the absorption even extended to the NIR region (Figure 3E). On the other hand, the valence band XPS spectrum showed that the position of valence band maximum (VBM) of the as-synthesized HTCC particles was +1.90 V [vs. normal hydrogen electrode (NHE)] (Figure 3C), whereas the Kubelka–Munk plot illustrated that the bandgap energy (E_*g*_) of HTCC particles was 1.2 eV (Figure 3D). By the following equation:

(1) $${\text{E}}_{\text{CB}}=E_{\text{VB}}-{\text{E}}_{\text{g}}$$

where E_*CB*_ and E_*VB*_ represent the potential of conduction band minimum (CBM) and VBM, respectively, and E_*g*_ represents the bandgap energy, the position of CBM of HTCC particles was determined to be +0.70 V (vs. NHE).

By converting the bandgap energy into wavelength using the equation:

(2) $$\lambda =1240/{\text{E}}_{\text{g}}$$

Hydrothermal carbonation carbon particles were predicted to be activated by radiation with a length short than 1,030 nm, which falls in the NIR region favoring the practical application with natural sunlight. Furthermore, HTCC particles were verified to be VL-responsive with photocurrent generation under 2,000 W m^–2^ VL irradiation (Figure 3F). Under the on–off cycles of VL irradiation, uniform and fast generation of photocurrent of 0.2 μA cm^–2^ by HTCC was observed. Hence, a hybrid system was constructed to enhance the BHP of *K. pneumoniae* with HTCC.

### Hydrogen Production Efficiency of Hydrothermal Carbonation Carbon–*K. pneumoniae* Hybrid System

**FIGURE 4:**
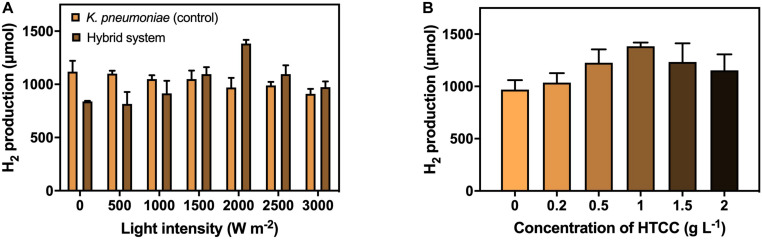
H_2_ production by the hybrid system **(A)** with 1 g L**^−^**^1^ HTCC in 3 h under different VL intensities (0–3,000 W m**^−^**^2^), and **(B)** under 2000 W m**^−^**^2^ VL irradiation in 3 h with different concentrations of HTCC (0–2 g L**^−^**^1^).

The H_2_ production under different VL intensities was performed to investigate the effect of VL on H_2_ production efficiency (Figure 4A). The result illustrated the 3-h H_2_ production with 1 g L^–1^ HTCC under VL irradiation of different intensities. The H_2_ production by the hybrid system increased at a low light intensity, peaked at a light intensity of 2,000 W m^–2^, and decreased with the increasing light intensity. Hence, the optimal light intensity for the hybrid system was considered to be 2,000 W m^–2^ with 1,380 μmol H_2_ production in 3 h. Higher intensity of VL could provide more photons for the activation of HTCC to generate e^–^–h^+^ pairs to participate in H_2_ production, therefore promoting the H_2_ production of the hybrid system when increased from 0 to 2,000 W m^–2^. However, the photocatalytic rate reached a plateau and became independent of VL intensity at moderate to high intensity because the recombination of e^–^–h^+^ pairs compete with the generation of e^–^–h^+^ pairs, and the rate is limited by the transferring electrons from the photocatalysts to surrounding substances (Mozia, 2010). In addition, it was also reported that high intensity of VL would exert phototoxicity and lower the viability of bacterial cells (Lipovsky et al., 2009). These two phenomena led to a reduction in H_2_ production in high VL intensity.

The concentration of photocatalyst is one of the factors affecting the efficiency of the photocatalytic reaction. Therefore, the addition concentration of catalyst on the H_2_ production efficiency was also studied under VL irradiation of 2,000 W m^–2^ (Figure 4B). The amount of H_2_ produced first increased with the concentration of HTCC and then reduced with the increasing high concentration of HTCC. The optimal HTCC concentration was found to be 1 g L^–1^, which produced 1,380 μmol H_2_ in 3 h. The elevation in H_2_ production efficiency could be explained by the increase in the total surface area available to receive the VL irradiation at low concentration (Kaneco et al., 2004; Galedari et al., 2019), and so, more e^–^-h^+^ pairs can be generated. Thus, more e^–^ would be available to participate in the enhancement of H_2_ production. However, when the concentration of HTCC was too high, the reaction mixture would become turbid, and the turbid reaction mixture would lead to the occurrence of scattering effect (Kaneco et al., 2004; Galedari et al., 2019), which may block the light penetration and scatter away from the light, and so, less radiation could reach the surface of HTCC in the bottom layer. With a higher concentration of HTCC, the agglomeration of particles would occur more easily and thus reduce the surface area subjected to light irradiation (Kaneco et al., 2004; Galedari et al., 2019). Therefore, less photogenerated e^–^–h^+^ pairs could take part in the enhancement of H_2_ production when HTCC concentration was too high because fewer HTCCs were photo-activated.

Under the optimized VL intensity and HTCC concentration, the H_2_ production efficiency of the hybrid system was enhanced by 35.3%. Compared to other organic-microbe hybrid system (Supplementary Table 5), the enhancement was similar to that of surface precipitated CdS-*E. coli* hybrid system (Wang et al., 2017) but far lower than that of TiO_2_-*E. coli* hybrid system (Ramprakash and Incharoensakdi, 2020). Despite the poorer enhancement, these hybrid systems required the use of cysteine as hole scavenger and/or methyl viologen as electron mediator which increased the cost of the operation. Furthermore, these hybrid systems utilized glucose instead of CG as carbon source which might not be capable of bioremediation of CG.

### Stability of HTCC-*K. pneumoniae* Hybrid System

**FIGURE 5:**
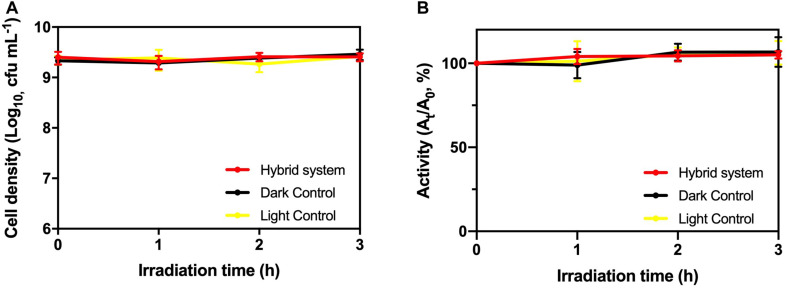
**(A)** Cell density and **(B)** TTC dehydrogenase activity of *K. pneumoniae* during 3-h H_2_ production under 2,000 W m^−2^ VL irradiation and control groups.

**FIGURE 6:**
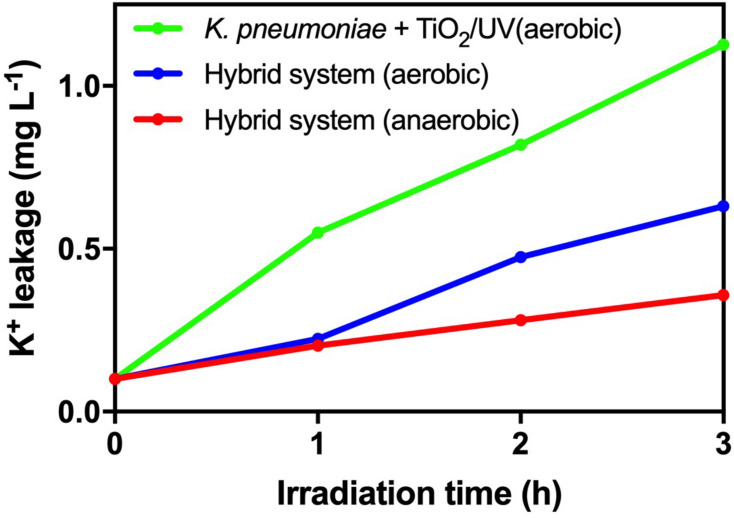
Leakage of intracellular K^+^ during 3-h H_2_ production of the hybrid system in anaerobic and aerobic conditions under 2,000 W m^−2^ VL irradiation and photocatalytic inactivation of *K. pneumoniae* with 1 g L^−1^ TiO_2_ under UV irradiation.

From Figure 5, there was no significant deviation in cell viability and TTC activity of *K. pneumoniae* in the hybrid system compared with light control and dark control. Therefore, the photogenerated e^–^–h^+^ pairs from HTCC did not induce changes in cell density and physiological condition of *K. pneumoniae*. On the other hand, intracellular K^+^ leakage was used as the indicator for cell membrane integrity because it occurs when the cell membrane is damaged and its permeability is affected (Cox et al., 2000). The leakage of intracellular K^+^ would increase the environmental K^+^ concentration. From Figure 6, the leakage of intracellular K^+^ of the hybrid system was far smaller than that of the hybrid system under aerobic condition and photocatalytic inactivation of *K. pneumoniae*. It is believed that the anaerobic condition can minimize the formation of reactive oxygen species (ROSs) by the photogenerated e^–^–h^+^ pairs. Therefore, *K. pneumoniae* cells of the hybrid system under anaerobic condition can maintain high integrity during H_2_ production.

As a result, based on cell viability, TTC activity and cell membrane integrity, it is believed that the hybrid system can remain stable during H_2_ production under anaerobic condition.

### Mechanism for Enhanced Hydrogen Production

#### Electron Generation and Transduction

**FIGURE 7:**
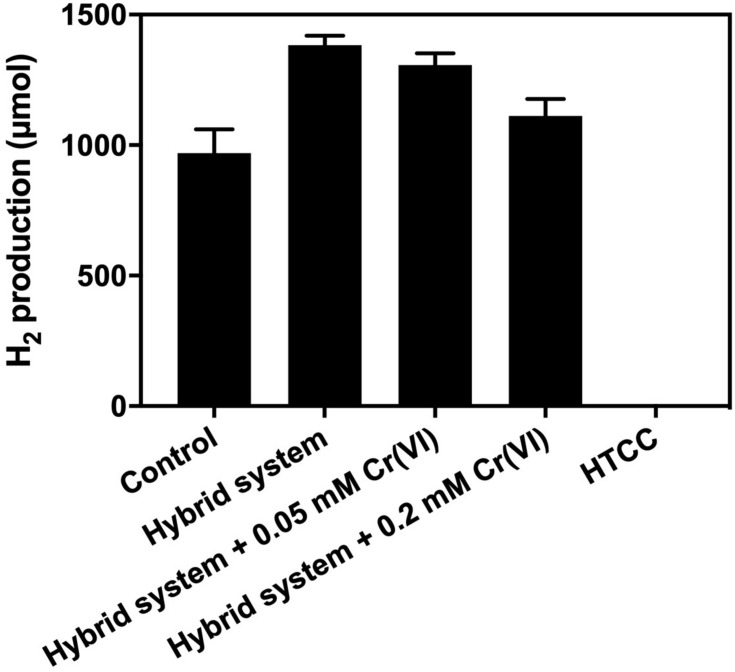
Result of electron scavenger study of the hybrid system under 2,000 W m**^−^**^2^ VL in irradiation 3 h.

From Figure 7, the addition of 1 g L^–1^ HTCC into an SBC medium supplemented with 20 g L^–1^ CG and 10% (v/v) methanol did not produce any H_2_ in 3 h. Hence, the additional H_2_ produced from the hybrid system under VL irradiation was not contributed by photocatalytic water splitting. This could be predicted from the band structure of HTCC that the CB potential of HTCC was +0.70 V (vs. NHE), which was not more negative than the reduction potential of H^+^/H_2_ (0 V vs. NHE), and so, the photogenerated e^–^ could not convert H^+^ into H_2_ directly.

Cr (VI) is commonly used as the electron scavenger to study the mechanism of photocatalysis (Wang et al., 2011), in which it would quench the photogenerated e^–^ during the 3-h H_2_ production. The result of the electron scavenger study showed that the amount of additional H_2_ production decreased with the increase in Cr (VI) concentration. The photogenerated e^–^ was removed by Cr (VI) and thus could not reach the *K. pneumoniae* cells to enhance the production of H_2_. Therefore, it is believed that H_2_ production of the hybrid system was enhanced by the photogenerated e^–^ from the HTCC, which was transferred to *K. pneumoniae* cells and interacted with the pathway of BHP.

#### Hydrogen Production Metabolic Pathway

**FIGURE 8:**
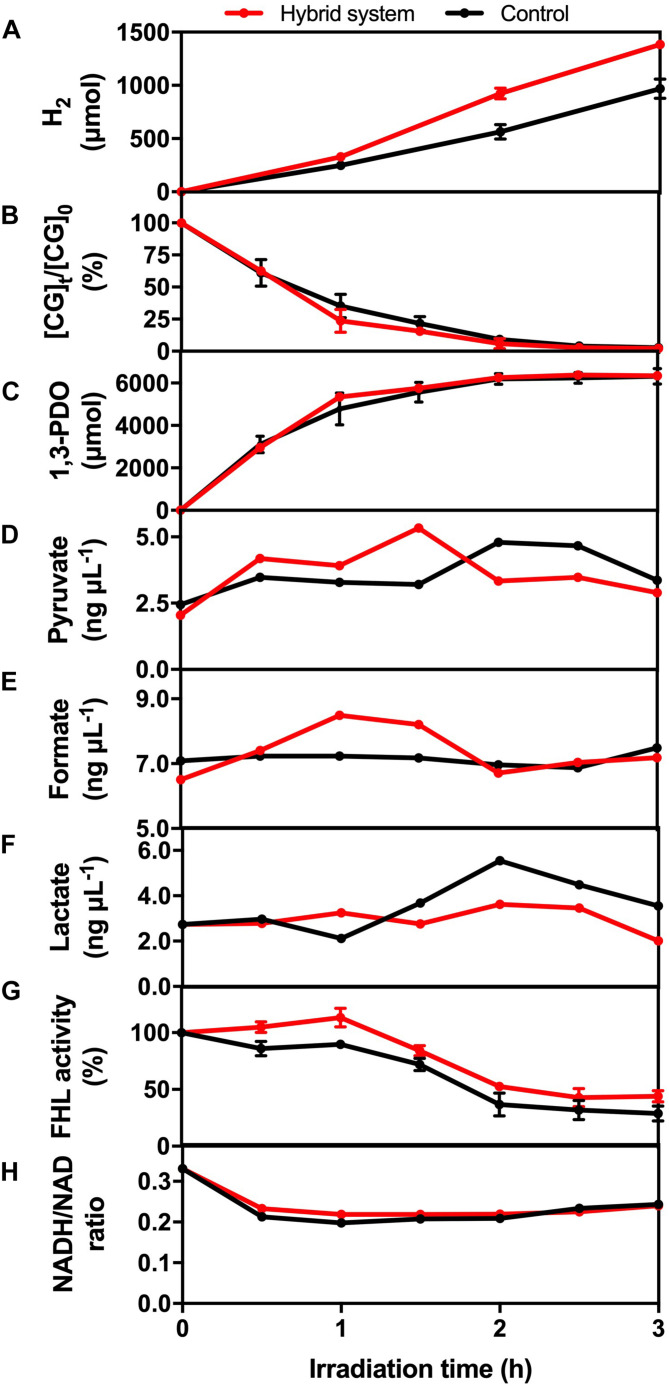
**(A)** H_2_ production, **(B)** CG utilization, **(C)** 1,3-PDO production, **(D)** pyruvate concentration, **(E)** formate concentration, **(F)** lactate concentration, **(G)** formate hydrogen lyase (FHL) activity, and **(H)** NADH/NAD ratio of the hybrid system under VL irradiation (2,000 W m^− 2^) and control.

The glycerol metabolism pathway of *K. pneumoniae* consists of oxidative and reductive dissimilation pathways (Streekstra et al., 1987; Zeng et al., 1993, 1996). For the oxidative pathway, glycerol is first metabolized to dihydroxyacetone by glycerol dehydrogenase and then is oxidized to pyruvate. Pyruvate is further converted into different metabolites such as H_2_. On the other hand, 3-hydroxypropionaldehyde is metabolized by glycerol dehydratase and further reduced to 1,3-PDO in the reductive pathway. Therefore, the change in levels of CG, intermediate and reducing equivalents, and the enzymatic activity of the hybrid system during the 3-h H_2_ production were monitored to reflect the mechanism under BHP (Figure 8).

Crude glycerol acts as the energy source and electron donor for *K. pneumoniae* to perform BHP. The utilization efficiency of CG would affect the concentration of different intermediates, such as pyruvate and formate, which was important for H_2_ production. From Figure 8B, the consumption rate of CG was similar in the first 30 min for both experimental groups and then was slightly faster in the hybrid system compared with the light control. One possible reason is that the photogenerated e^–^ could accelerate the conversion of CG by *K. pneumoniae*, and so, CG diffused into the cells more quickly.

1,3-PDO is the final product formed in the reductive pathway. From Figure 8C, the production rate of 1,3-PDO was similar in the first 30 min, whereas the rate was slightly faster in the hybrid system compared with the light control from 30 to 90 min, and finally, the total production of 1,3-PDO was the same. The faster production of 1,3-PDO from 30 to 90 min could be explained by the faster utilization of CG at the same time interval, indicating that some of the CG consumed by *K. pneumoniae* would still be metabolized to 1,3-PDO, and this pathway was not inhibited by the photogenerated e^–^.

Pyruvate and formate are the two intermediates involved in the downstream H_2_ production pathway of CG by *K. pneumoniae*. Figure 8D shows that the intracellular pyruvate concentration increased faster in the hybrid system compared with the control in the first 90 min. The photogenerated e^–^ from HTCC may be involved to accelerate the pathway of conversion of CG into pyruvate. Therefore, there would be more pyruvate available for further conversion into formate, which was illustrated by the higher formate concentration in the hybrid system in the first 90 min compared with the control (Figure 8E). The additional formate synthesized in the hybrid system could be used as the substrate for FHL to produce additional H_2_ (Figure 8A). The drop in intracellular formate in the hybrid system after 90 min was probably correlated with the decrease in intracellular pyruvate, as the consumption rate of CG by the hybrid system was slowed down.

Lactate fermentation is another process consuming pyruvate (Abdel-Rahman et al., 2013), which leads to the reduction in H_2_ production. Figure 8F shows that the intracellular lactate concentration was similar in both the hybrid system and the control. Therefore, it proved that the elevated pyruvate in the hybrid system was transformed into formate instead of lactate in the first 90 min. Moreover, the intracellular lactate level did not fluctuate much in the hybrid system, whereas the accumulation of lactate was found in the control in the last 90 min. It indicated that lactate production was inhibited in the hybrid system, and so, more pyruvate can be reserved for the production of H_2_.

Besides the intracellular intermediates, the activity of FHL was also important for H_2_ production because it can produce H_2_ through the decomposition of formate in anaerobic conditions (Bagramyan et al., 2002). Hence, the higher activity of FHL could enhance the H_2_ production of *K. pneumoniae* by cleaving the formate more quickly and thus directing the metabolic flux toward the pathway of H_2_ production. Figure 8G shows that FHL activity was enhanced in the hybrid system throughout the whole H_2_ production experiment, and thus, it accounted for the additional H_2_ produced by the hybrid system.

Several studies have reported that NADH can participate in H_2_ production through the formate pathway or the NADH pathway (Zeng et al., 1996; Niu et al., 2011). On the other hand, metabolic flux and thus the H_2_ production were also affected by the intracellular redox potential (Liu et al., 2013), which can be monitored with the intracellular NADH/NAD ratio. Figure 8H shows that the NADH/NAD ratio was slightly higher in the hybrid system. The photogenerated e^–^ could induce the faster regeneration of NADH and thus elevate the reduction potential inside the bacterial cells. The higher level of NADH and reduction potential are favorable for BHP in the hybrid system.

**SCHEME 1:**
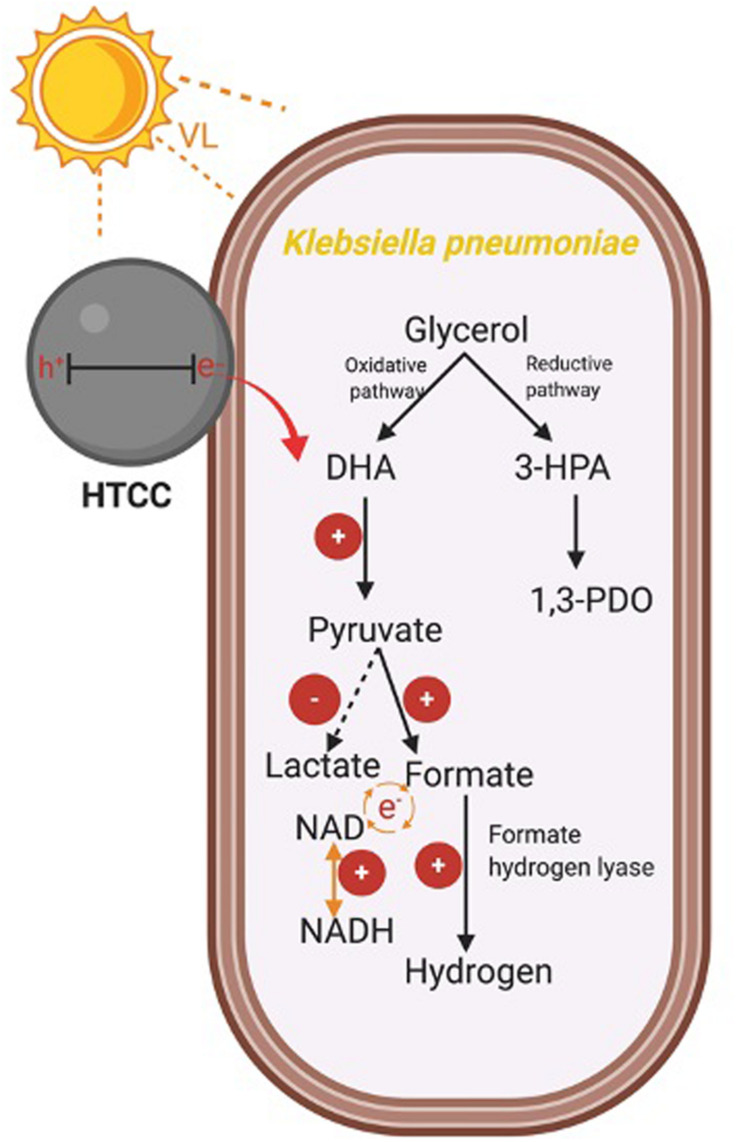
Mechanism of the enhanced H_2_ production in the hybrid system.

In summary, the mechanism for enhanced hydrogen production was summarized in Scheme 1. The photogenerated e^–^ from HTCC could promote the oxidative pathway of glycerol, leading to the higher production of pyruvate. Besides, the pyruvate was effectively transformed to formate for hydrogen production under the assistance of e^–^, whereas the conversion of lactate was suppressed with the injection of e^–^. The enhanced concentration of formate finally led to the enhanced production of hydrogen with the increased activity of FHL. Besides, due to the important role of NADH/NAD, the elevated NADH/NAD ratio also favored the hydrogen production in the hybrid system in a subtle manner.

## Conclusion

Presently, *K. pneumoniae* with CG-utilizing and H_2_ production ability was successfully isolated and purified from RAS. The CG concentrations in the H_2_ production strategy for *K. pneumoniae* were optimized to be 20, 50, and 20 g L^–1^ for bacterial cultivation, hydrogenase induction, and H_2_ production, respectively. Under the optimized condition, 3-h BHP of *K. pneumoniae* can reach 1,020 μmol. On the other hand, VL-active HTCC microspheres were successfully synthesized by hydrothermal reaction to construct an organic–microbe hybrid system. With the optimized VL intensity at 2,000 W m^–2^ and HTCC concentration at 1 g L^–1^, the hybrid system can produce 1,380-μmol H_2_, achieving 35.3% enhancement in BHP. Based on the electron scavenger study, the photogenerated e^–^ interacted with *K. pneumoniae* cells to produce additional H_2_ instead of directly reducing water to H_2_. By the quantification of intermediates, enzymatic activity, and reducing equivalents in the BHP pathway, the metabolic flux of the hybrid system shifted toward H_2_ production, which is believed to be caused by the photogenerated e^–^. Hence, this organic–microbe hybrid system can serve as a waste-to-energy system for bioconversion of CG in H_2_ under VL irradiation.

## Data Availability Statement

The original contributions presented in the study are included in the article/Supplementary Material, further inquiries can be directed to the corresponding authors.

## Author Contributions

HC did the experiments. KX worked together in bacterial strain isolation and purification. All authors did manuscript ideas and editing.

## Conflict of Interest

The authors declare that the research was conducted in the absence of any commercial or financial relationships that could be construed as a potential conflict of interest.
