# Supplementary material for: Bioremediation of Crude Glycerol by a Sustainable Organic–Microbe Hybrid System
Source: Front Microbiol. 2021 Apr 8;12:654033. doi: 10.3389/fmicb.2021.654033 (PMC8103898; doi:10.3389/fmicb.2021.654033)
Supplement: Supplementary file 1 [file Data_Sheet_1.docx]

Supplementary Materials

Bioremediation of crude glycerol by a sustainable organic-microbe hybrid system

Ho Shing Chan^1,+^, Kemeng Xiao^1,+^, Tsz Ho Tsang^1^, Cuiping Zeng^2^, Bo Wang^2,*^, Xingxing Peng^3^, Po Keung Wong^1,4*^

^1^ School of Life Sciences, The Chinese University of Hong Kong, Hong Kong SAR, China

^2^ CAS Key Laboratory of Quantitative Engineering Biology, Shenzhen Institute of Synthetic Biology, Shenzhen Institutes of Advanced Technology, Chinese Academy of Sciences, Shenzhen, China

^3^ School of Environmental Science and Engineering, Sun Yat-sen University, Guangzhou, China

^4^ Institute of Environmental Health and Pollution Control, Guangdong University of Technology, Guangzhou, China

**^+^Equal contribution**

*** Corresponding authors:**

Bo Wang

bo.wang@siat.ac.cn

Po Keung Wong
pkwong@cuhk.edu.hk


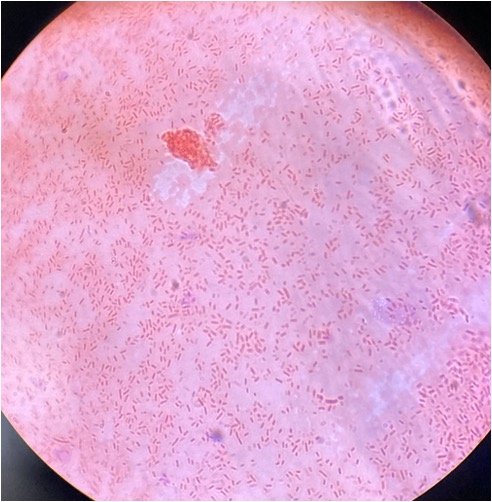


Figure S1. Morphology of Gram-stained isolated bacterial strain (1,000X).


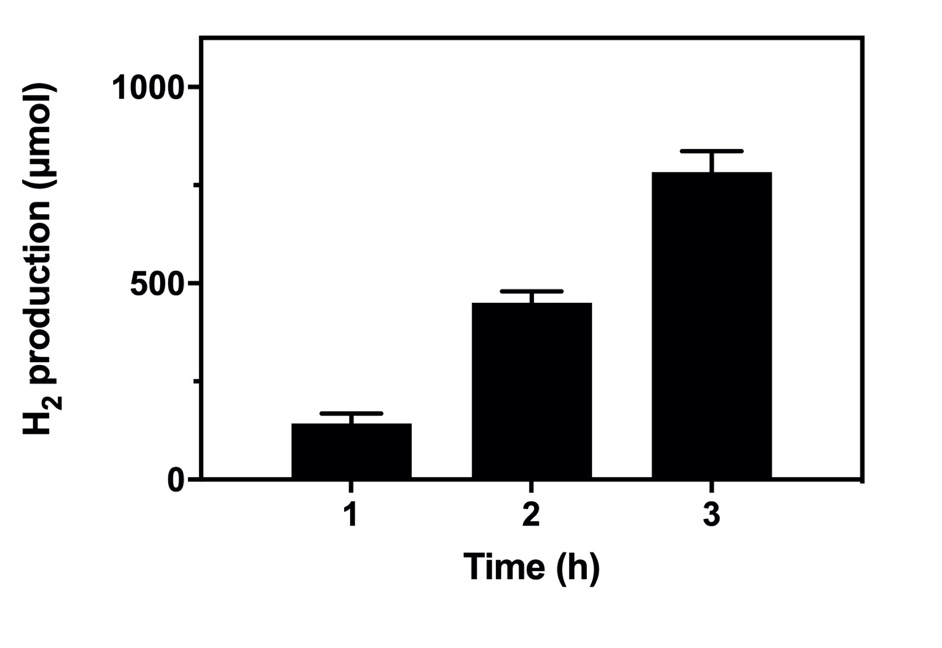


**Figure S2.** H_2_ production efficiency of the isolated bacterial strain.

Table S1. Composition of the CG supplied by Champway Technology Limited.

| Components | Average percentage (%, w/w) |
| --- | --- |
| Glycerol | 74.4 |
| Methanol | 1.2 |
| Moisture | 1.1 |
| Non-glycerol organic impurities | 11.5 |
| Soluble inorganic salt (Na_2_SO_4_) | 8.3 |
| Insoluble substances | 3.5 |

Table S2. Composition of MOPS minimal medium.

| Component | Final concentration (g L^-1^) |
| --- | --- |
| MOPS | 8.37 |
| Tricine | 0.717 |
| FeSO_4_·7H_2_O | 0.0278 |
| NH_4_Cl | 0.509 |
| K_2_SO_4_ | 0.0481 |
| CaCl_2_·2H_2_O | 0.0735 |
| MgCl_2_·6H_2_O | 0.106 |
| NaCl | 2.92 |
| K_2_HPO_4_ | 0.230 |
| (NH_4_)_6_Mo_7_O_24_·4H_2_O | 3.71×10^-6^ |
| H_3_BO_4_ | 2.47×10^-5^ |
| CoCl_2_·6H_2_O | 7.14×10^-6^ |
| CuSO_4_ | 2.50×10^-6^ |
| MnCl_2_·4H_2_O | 1.58×10^-5^ |
| ZnSO_4_·7H_2_O | 2.88×10^-6^ |

**Table S3.** Composition of BC medium and SBC medium.

| Component | Final concentration (g L^-1^) | |
| --- | --- | --- |
|  | BC | SBC |
| (NH_4_)2HPO4 | 10 | 10 |
| K2SO4 | 2 | 2 |
| NaCl | 0.3 | 0.3 |
| MgSO4·7H2O | 0.2 | / |
| FeSO4·7H2O | 4×10^-3^ | / |
| ZnSO4·7H2O | 9×10^-4^ | / |
| CuSO4·5H2O | 4×10^-4^ | / |
| MnSO4·H2O | 2×10^-4^ | / |
| CaCl2·2H2O | 8×10^-4^ | / |
| Na2B4O_7_·10H2O | 9×10^-5^ | / |
| Na2SeO3·5H2O | 6×10^-4^ | / |
| (NH4)6Mo7O24·4H_2_O | 4.25×10^-4^ | / |
| (NH4)2Ni(SO4)2·6H2O | 9×10^-4^ | / |

Table S4. General characteristics and results of identification of bacterial strain 2C.

|  |  | Observations | SIM index*/Percentage identity** |
| --- | --- | --- | --- |
| General characteristic | Microscopic morphology | Rod shape | / |
|  | Gram staining | Negative | / |
| Identification method | Fatty acid profiling | *Klebsiella pneumoniae* | 0.663 |
|  | 16S rRNA sequencing | *Klebsiella pneumoniae* | 100% |

* Similarity (SIM) index represents the similarity between sample and the library, the higher SIM index indicated a better matching. The maximum value and the threshold value for a significant match are 1.000 and 0.500.

** Result of gene sequencing matched with BLASTn sequence database of National Centre for Biotechnology Information.

Table S5. H_2_ production by organic/inorganic-microbe hybrid systems.

| Inorganic-microbe hybrid systems | Carbon source | Additional chemicals | Enhancement in H_2_ production | Reference |
| --- | --- | --- | --- | --- |
| HTCC-*K. pneumoniae* | Crude glycerol | - | 35.3% | This study |
| Surface precipitated CdS-*E. coli* | Glucose | Cysteine (hole scavenger) | ~30% | Wang et al., 2017 |
| TiO_2_-*E. coli* | Glucose | Cysteine (hole scavenger), Methyl viologen (electron mediator) | ~150% | Ramprakash and Incharoensakdi, 2020 |
